# Kisspeptin-10 Induces β-Casein Synthesis via *GPR54* and Its Downstream Signaling Pathways in Bovine Mammary Epithelial Cells

**DOI:** 10.3390/ijms18122621

**Published:** 2017-12-05

**Authors:** Jianhua Sun, Juxiong Liu, Bingxu Huang, Xingchi Kan, Guangxin Chen, Wei Wang, Shoupeng Fu

**Affiliations:** College of Animal Science and Veterinary Medicine, Jilin University, Changchun 130062, China; jhsun15@mails.jlu.edu.cn (J.S.); juxiong@jlu.edu.cn (J.L.); huangbingxu16@mails.jlu.edu.cn (B.H.); kanxc16@mails.jlu.edu.cn (X.K.); chengx15@mails.jlu.edu.cn (G.C.); wangwei@jlu.edu.cn (W.W.)

**Keywords:** Kisspeptin-10, GPR54, β-casein

## Abstract

Kisspeptins (Kps) play a key role in the regulation of GnRH axis and as an anti-metastasis agent by binding with *GPR54*. Recently, we observed that the expression of *GPR54* was higher in the lactating mammary tissues of dairy cows with high-quality milk (0.81 ± 0.13 kg/day of milk protein yield; 1.07 ± 0.18 kg/day of milk fat yield) than in those with low-quality milk (0.51 ± 0.14 kg/day of milk protein yield; 0.67 ± 0.22 kg/day of milk fat yield). We hypothesized that Kp-10 might regulate the milk protein, β-casein (CSN2) synthesis via GPR54 and its downstream signaling. First, we isolated the bovine mammary epithelial cells (bMECs) from lactating Holstein dairy cows, and treated them with different concentrations of Kp-10. Compared with the control cells, the synthesis of CSN2 is significantly increased at a concentration of 100 nM of Kp-10. In addition, the increased effect of CSN2 synthesis was blocked when the cells were pre-treated with the selective inhibitor of GPR54 Peptide-234 (P-234). Mechanistic study revealed that Kp-10 activated ERK1/2, AKT, mTOR and STAT5 in bMECs. Moreover, inhibiting ERK1/2, AKT, mTOR and STAT5 with U0126, MK2206, Rapamycin and AG490 could block the effects of Kp-10. Together, these results demonstrate that Kp-10 facilitates the synthesis of CSN2 via GPR54 and its downstream signaling pathways mTOR, ERK1/2, STAT5 and AKT.

## 1. Introduction

As an important nutritional source, milk protein plays an important role in human life; casein and whey protein are the primary sources of milk protein [1]. The reduction of milk quality is an important issue for milk industry, and milk protein, especially β-casein (CSN2), is the benchmark of milk quality. Milk protein synthesis is influenced by environmental elements, hormones and growth factors. The levels of hormones and growth factors vary along the pregnancy lactation cycle [2,3]. Many factors, especially protein hormones regulate the synthesis of milk protein, and looking for protein hormones that regulate the synthesis of milk protein has become the focus of recent research. According to the reports, growth hormone (GH) in calves contributes to the growth and development of the mammary gland and thereafter to increase milk production [4,5]. In addition, the protein hormones ghrelin (AG) and unacylated ghrelin (UAG) can induce the synthesis of milk protein CSN2 in bovine mammary epithelial cells [6]. Therefore, the protein hormones that regulate the milk protein CSN2 may play an important role in improving the quality of milk in production.

Kisspeptins (Kps), products of the *Kiss-1* gene, bind to a G protein-coupled receptor known as GPR54. These short peptides have many biological forms, such as Kp-10, Kp-13, Kp-14 and Kp-54 [7]. Kp-10 has become a common choice for the current *Kiss-1*/GPR54 system functions [8,9,10]. Based on recent reports the *Kiss-1*/GPR54 system has important roles in the reproductive axis, anti-metastasis, and the regulation of hormone secretion in mammals [8,11,12]. Kp-10 is also produced by the hypothalamus which can regulate the synthesis of oxytocin that is essential for normal birth and lactation [13]. Additionally, Kp-10 can stimulate luteinizing hormone (LH) and GH release in rats, cows, and sheep [14]. Among these effects of Kp-10, oxytocin plays an important role in the process of lactation [9], suggesting that Kp-10 may also play a role in the synthesis of milk protein. LH is also linked to stimulation of milk production in mammals [15]. Moreover, GH regulates mammary gland growth and development, which also contributes to milk protein synthesis [5]. This data together implicates Kp-10 as a key factor in the synthesis of milk protein. In addition, in the central nervous system PRL inhibits the production of Kisspeptin; however, in the presence of high concentrations of PRL in menstrual blood from lactating females, Kisspeptin concentrations in the peripheral blood maintained normal levels [16]. Thus, Kisspeptin not only acts in the central nervous system, but also in the periphery, and this effect may be closely related to the process of lactation. Other studies have shown the expression of *Kiss-1*/GPR54 in breast tissue [8,17], and our study found that the expression of GPR54 was strongly positively correlated with CSN2 in mammary gland tissue, suggesting that *Kiss-1*/GPR54 may facilitate the synthesis of CSN2 in breast tissue.

Milk protein synthesis is a complex biological process that requires the participation of a variety of signaling pathways, and CSN2 synthesis is just as complex. Studies have shown that the main signals involved in the process of CSN2 synthesis include the mTOR and JAK2/STAT5 pathways [18,19,20,21]. The transcription factor STAT5 is important for CSN2 synthesis, because the expression of the CSN2 gene can be enhanced by STAT5 [22]. In addition, STAT5 is of great significance to the occurrence and development of the mammary gland, and is essential for lactation and milk protein synthesis [23,24]. Some studies have shown that the PI3K/AKT pathway is involved in lipid lactose milk protein synthesis, and furthermore, that there is a direct link between PI3K/AKT and STAT5 [25,26]. Moreover, ERK1/2 and AKT are also both involved in the process of CSN2 synthesis [6]. Among them, thePI3K/AKT and ERK1/2 pathways are both activated in mammary epithelial cell (MEC) number and their activity affected by IGF-I [27]. Notably, some studies have shown that Kp-10 can stimulate the ERK1/2 signaling pathway in the process of inhibiting the development of cancer and the initiation of puberty via GPR54 [8,28,29]. Prior to puberty, Kisspeptin and LHRH are influenced by mTOR and AKT [30], and STAT5 signaling is also involved in regulating the timing of puberty in kisspeptin cells [31]. Based on this data, we hypothesized that Kp-10 could modulate the synthesis of CSN2 by GPR54 and its downstream pathways STAT5, mTOR, ERK1/2 and AKT. Therefore, the present study serves as further evidence for the role of GPR54 and its downstream signals in the regulation of CSN2 synthesis.

## 2. Results

### 2.1. The Expression of β-Casein(CSN2) and GPR54 in High-Quality Milk and Low-Quality Milk Mammary Gland Tissues from Lactating Holstein Dairy Cows

**Figure 1:**
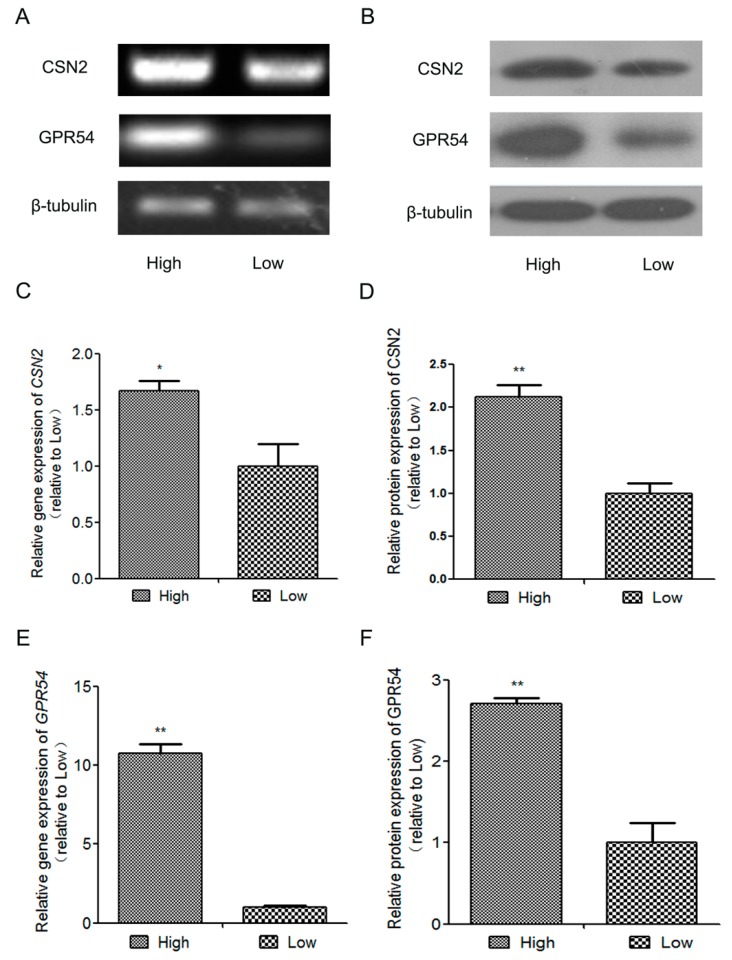
The gene and protein expressions of *β-casein* (*CSN2*) and *GPR54* in high-quality milk (High) and low-quality milk (Low) mammary gland tissues from lactating Holstein dairy cows. (**A**,**C**,**E**) The gene expressions of *GPR54* and *CSN2* were detected by PCR. (**B**,**D**,**F**) The protein expressions of GPR54 and CSN2 were detected by Western blot. The relative mRNA level was normalized to *β-tubulin* mRNA, and the relative protein levels were quantified by scanning densitometry and normalized to β-tubulin. The data are presented as the means ± S.D. (*n* = 5 per treatment; * *p* < 0.05, ** *p* < 0.01 vs. low-quality milk mammary gland tissues).

No previous study has elucidated the relationship between Kp-10/GPR54 and CSN2 in lactating dairy cows, so we undertook to fill this gap. The high-quality milk and low-quality milk mammary gland tissues were taken from lactating Holstein dairy cows (lactation = 210 ± 24 days). As shown in Figure 1, the mRNA and protein expression of *CSN2* and *GPR54* were both higher in high-quality milk than in low-quality milk mammary gland tissues (*n* = 5 per treatment, *p* < 0.05). The protein expression of GPR54 was strongly positively correlated with CSN2 (high-quality milk mammary gland tissues group *r* = 0.982987; low-quality milk mammary gland tissues group *r* = 0.909759). These results suggest that the activation of GPR54 might be connected with the expression of CSN2.

### 2.2. BMECs Culture and Identification

**Figure 2:**
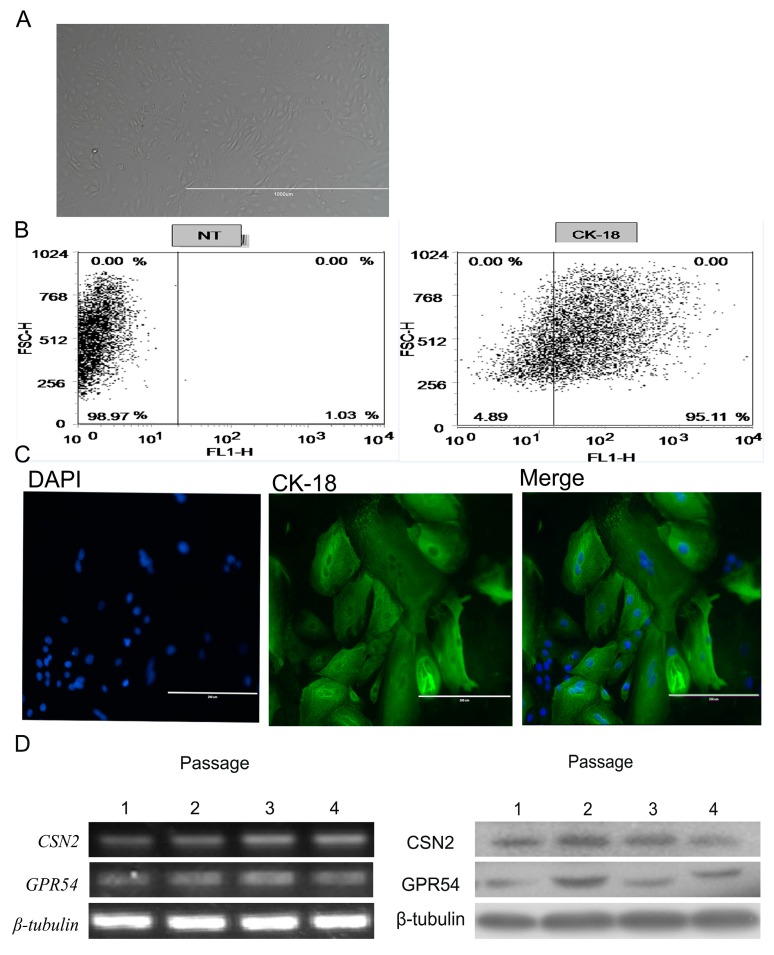
Bovine mammary epithelial cells (bMECs) culture and identification. (**A**) Morphology of cultured bMECs. Cells show the typical islands morphology of epithelial cells (bar = 200 μm); (**B**) The expression of CK-18 was detected by flow cytometry in bMECs; (**C**) Immunofluorescence labeling of CK-18 in bMECs (200× magnification) (bar = 200 μm); (**D**) Gene and protein expression of *CSN2* and *GPR54* in cultured bMECs (passages 1–4) (*n* = 3).

The gene and protein expression of *GPR54* and *CSN2* was higher in the high-quality milk than low-quality milk tissues, so we next wanted to determine whether the activation of GPR54 could induce the production of CSN2. We isolated the bMECs from lactating Holstein dairy cows. First, we observed the morphology (Figure 2A) and the expression of CK-18 to ensure the purity of bMECs before each experiment. The purity of the isolated cells was up to 95.11% CK-18 expression (Figure 2B,C). Next, we evaluated the gene and protein expression of *CSN2* and *GPR54* in cultured bMECs from passage 1 to passage 4. The results indicate that genes and proteins of *GPR54* and *CSN2* were expressed at passages 1–4 (Figure 2D). These results proved that the following experiments could be continued.

### 2.3. Kp-10 Promoted CSN2 mRNA and Protein Expression in Bovine Mammary Epithelial Cells (bMECs)

**Figure 3:**
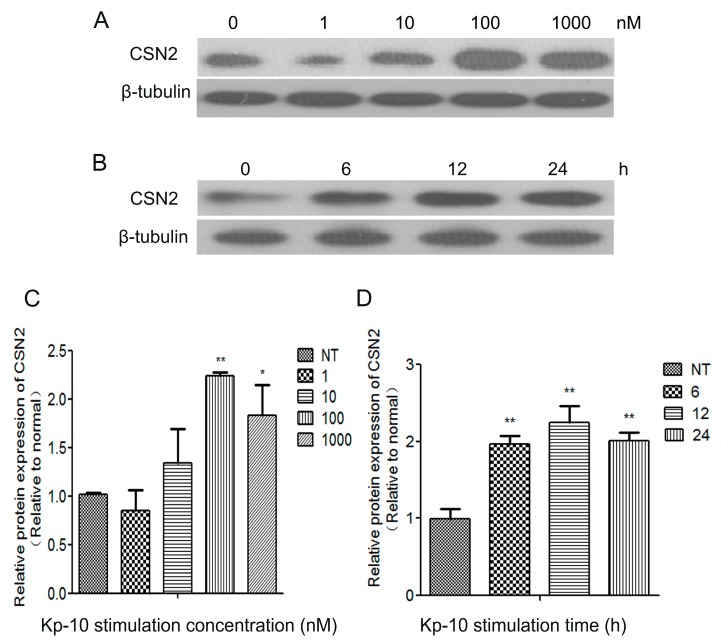
Kp-10 promoted CSN2 protein expression in bMECs. (**A**,**C**) Cells were incubated for 12 h with different concentrations of Kp-10 (0, 1, 10, 100 and 1000 nM); (**B**,**D**) cells were incubated with 100 nM Kp-10 for different periods (0, 6, 12 and 24 h), and protein expression of CSN2 was examined by Western blot; (**C**,**D**), the relative protein levels were quantified by scanning densitometry and normalized to β-tubulin. The data is presented as the means ± S.D. (*n* = 3; * *p* < 0.05, ** *p* < 0.01 vs. non-treated (NT) control group).

Kp-10 has been shown to stimulate the secretion of growth hormone and prolactin directly from cultured bovine anterior pituitary cells through GPR54, and Kp-10 stimulates the secretion of GH and PRL from cultured bovine AP cells [11,12,32]. In addition, GH and PRL are vital to the process of lactation and milk protein synthesis. However, little is known about whether Kp-10/GPR54 regulates CSN2 synthesis in bMECs. To elucidate that, we detected the protein expression of CSN2 in cells treated with different concentrations of Kp-10 for 12 h (the bMECs were starved for 4 h before each experiment). The results show that 100 nM Kp-10 significantly increased the protein expression of CSN2 at 12 h compared to the control cells (NT) (*p* < 0.05; Figure 3A,C). Then, we detected the protein expressions of CSN2 treated with 100 nM Kp-10 for 0, 6, 12 and 24 h. The Western blot result illustrates that the protein expression of CSN2 was obviously increased after 12 h treatment with 100 nM Kp-10 in bMECs (*p* < 0.01; Figure 3B,D). This data further supports our conjecture that Kp-10 induces the protein expression of CSN2 in bMECs. Considering the above results, 100 nM Kp-10 was selected as the best concentration to use for the subsequent experiments.

### 2.4. Peptide-234 Inhibited the Kp-10-Induced mRNA and Protein Expression of CSN2 in BMECs

**Figure 4:**
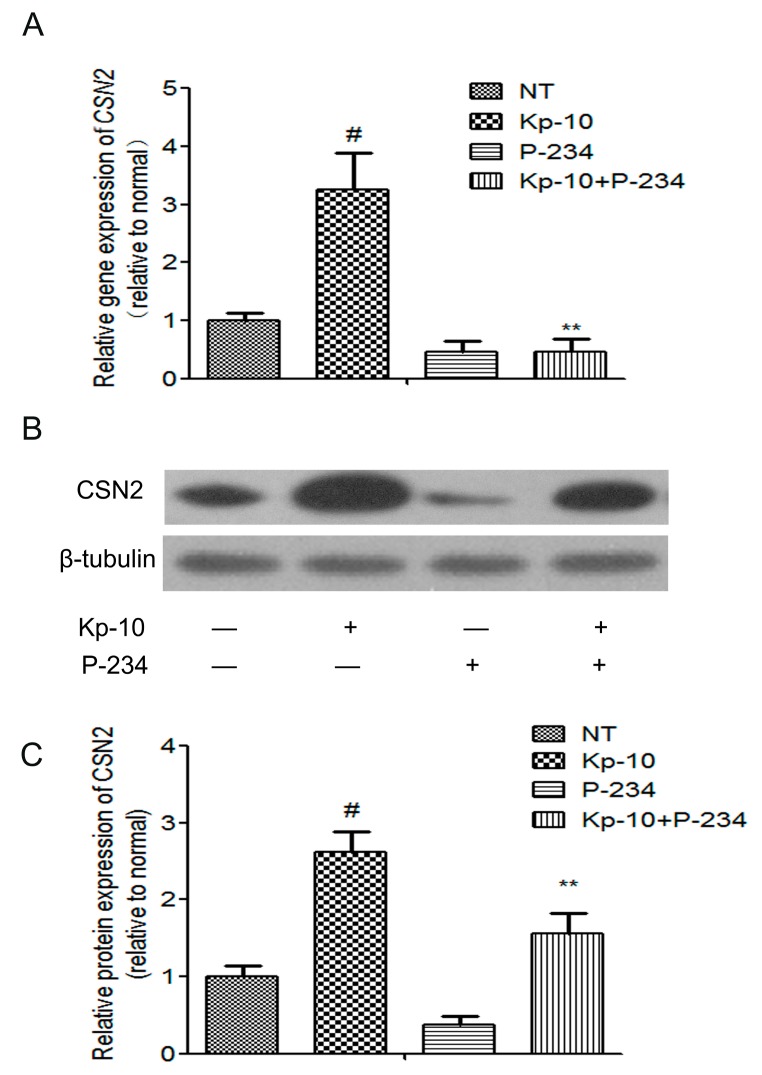
Peptide-234 (P-234) inhibited the Kp-10-induced mRNA and protein expression of *CSN2* in bMECs. The cells were pre-treated for 1 h with P-234 (1 μM) and then incubated for 12 h with Kp-10 (100 nM). The gene (**A**) and protein (**B**,**C**) expression of *CSN2* was examined by quantitative real-time PCR and Western blot. The relative mRNA level was normalized to *β-tubulin* mRNA, and the relative protein levels were quantified by scanning densitometry and normalized to β-tubulin. The data are presented as the means ± S.D. (*n* = 3; ^#^ *p* < 0.01 vs. NT; ** *p* < 0.01 vs. Kp-10 treatment).

In a variety of mammals, Kp-10 plays an active role in the reproductive axis by combining its receptor GPR54 and stimulating gonadotropin releasing hormone neuron activity [33,34]. To investigate whether Kp-10 increased the mRNA and protein expressions of *CSN2* via GPR54, bMECs were pre-treated with Peptide-234 (a selective inhibitor frequently used to examine the function of GPR54) for 1 h in the presence or absence of 100 nM Kp-10. As shown in Figure 4, pre-treatment with P-234 blocked the Kp-10-induced mRNA expression of *CSN2* (Figure 4A) and protein expression (Figure 4B,C) in bMECs. These results provide further evidence that Kp-10 increased the synthesis of CSN2 in bMECs via GPR54.

### 2.5. Kp-10 Phosphorylates mTOR, ERK1/2, STAT5 and AKT in BMECs

**Figure 5:**
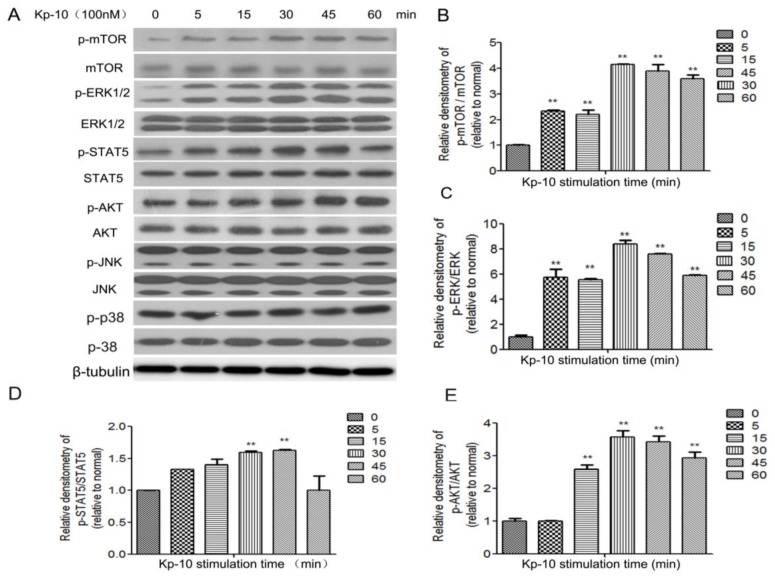
Kp-10 phosphorylates mTOR, ERK1/2, STAT5 and AKT in bMECs. BMECs were serum-starved for 4 h before treatment with Kp-10 (100 nM) over 60 min. (**A**) At the indicated timepoints, cell lysates were prepared and subjected to Western blot to detect the expression of MAPKs, mTOR, STAT5 and AKT. The phosphorylation ratio of mTOR (**B**); ERK1/2 (**C**); STAT5 (**D**); and AKT (**E**) was quantified. The ratio of the control group band (Kp-10 no treatment) was set to 1.00. The data is expressed as the means ± S.D. (*n* = 3; ** *p* < 0.01 vs. NT).

P38, JNK, mTOR, ERK1/2, STAT5 and AKT are associated with the milk protein synthesis process. To elucidate the downstream signaling mechanism through which Kp-10 modulates the synthesis of CSN2, we examined the effect of Kp-10 on the phosphorylation of mTOR, ERK1/2, STAT5 and AKT in bMECs. After a 4 h starvation, the cells were treated with 100 nM Kp-10 over 60 min. The ERK1/2 and AKT signaling pathways were significantly activated, while no significant changes were observed in the phosphorylation of p38 and JNK (Figure 5).

### 2.6. The mTOR, ERK1/2, STAT5 and AKT Inhibitors Attenuated the Kp-10-Induced Gene and Protein Expression of CSN2 in BMECs

**Figure 6:**
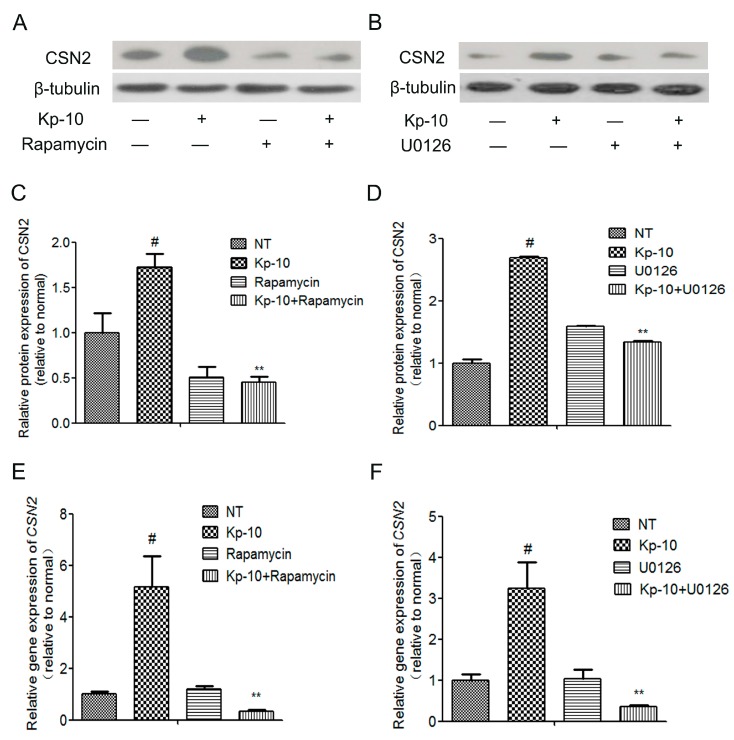
The mTOR inhibitor Rapamycin and the ERK1/2 inhibitor U0126 attenuated the Kp-10-induced the increased gene and protein expression of *CSN2* in bMECs. The cells were pre-treated for 1 h with Rapamycin (200 nM) or U0126 (10 μM) and then incubated for 12 h with Kp-10 (100 nM). The protein (**A**–**D**) and gene (**E**,**F**) expression of *CSN2* was examined by Western blot and quantitative real-time PCR. The data is presented as the means ± S.D. (*n* = 3; ^#^ *p* < 0.01 vs. NT; ** *p* < 0.01 vs. Kp-10 treatment).

**Figure 7:**
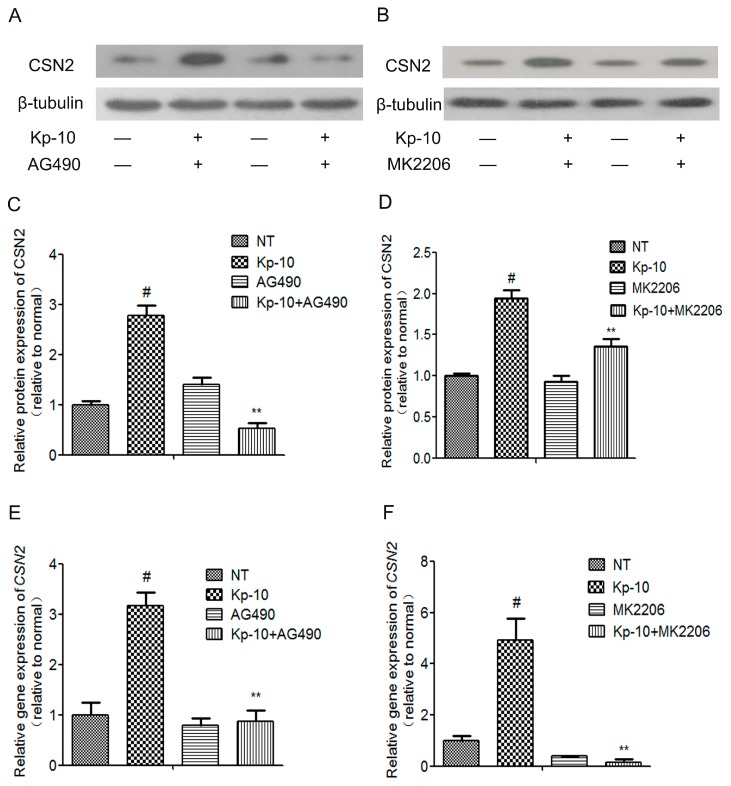
The STAT5 inhibitor AG490 and the AKT inhibitor MK2206 attenuated the Kp-10-induced increased the gene and protein expression of *CSN2* in bMECs. The cells were pre-treated for 1 h with AG490 (50 μM) or MK2206 (10 μM) and then incubated for 12 h with Kp-10 (100 nM). The protein (**A**–**D**) and gene (**E**,**F**) expression of *CSN2* was examined by Western blot and quantitative real-time PCR. The data is presented as the means ± S.D. (*n* = 3; ^#^ *p* < 0.01 vs. NT; ** *p* < 0.01 vs. Kp-10 treatment).

To determine whether the mTOR, ERK1/2, STAT5 and AKT signaling pathways mediated the regulation of CSN2 by Kp-10, we used ERK1/2-specific (U0126), AKT-specific (MK2206), mTOR-specific (Rapamycin) and STAT5-specific (AG490) inhibitors (dissolved in dimethylsulfoxide). The bMECs were pre-treated with the inhibitors for 1 h followed by cotreatment with 100 nM Kp-10 for 12 h. Quantitative real-time PCR was used to analyze the expression of *CSN2* mRNA levels. Western blot was used to analyze the protein expression of CSN2. As shown in Figure 6 and Figure 7, U0126, MK2206, Rapamycin and AG490 all significantly attenuated the gene and protein expression of *CSN2*. Taken together, these results suggest that the four signaling pathways, ERK1/2, AKT, mTOR and STAT5, all contribute significantly to the regulation of *CSN2* mRNA and protein expression induced by Kp-10.

## 3. Discussion

Altogether, our findings indicate that Kp-10 increased CSN2 synthesis via GPR54 and its downstream signaling pathways mTOR, ERK1/2 STAT5 and AKT in bMECs. The expression of GPR54 was higher in high-quality milk than low-quality milk tissues from lactating dairy Holstein cows, and the expression of CSN2 is strongly correlatedtoGPR54 in the tissues. The effect of Kp-10 on CSN2 synthesis in bMECs was blocked by pre-treatment with the GPR54 inhibitor P-234. In addition, the ERK1/2 inhibitor U0126, the mTOR inhibitor Rapamycin, the STAT5 inhibitor AG490 and the AKT inhibitor MK2206 attenuated the Kp-10-induced synthesis of CSN2 in bMECs. Therefore, we found that Kp-10 promotes the synthesis of CSN2 in bovine mammary epithelial cells by activating GPR54 and its downstream signaling partners of mTOR, STAT5, AKT and ERK1/2.

Milk protein synthesis is affected by a variety of factors, such as environments, the hormones, and genes, and requires the process of pregnancy and lactation [2]. Milk and milk products are important sources of nutrition for humans, globally [35]. Because milk protein is one of the main sources of human intake of animal protein, in recent years, research into milk protein synthesis and mechanisms has been gaining visibility. Some studies have focused on the effects of the functional integrity of the mammary gland and hormone-related proteins on the synthesis of milk proteins [36]. In addition, research into the effects of hormones in the regulation of milk protein synthesis, especially β-casein (CSN2), has become popular. One study in particular reported that Kp-10 could regulate the prolactin neurons in the late stage of pregnancy in bovine anterior pituitary cells [12]. Therefore, we hypothesized that Kp-10/GPR54 may be connected to milk protein synthesis, especially CSN2 synthesis.

The present study serves as evidence that GPR54 is expressed and activated in the mammary glands of lactating Holstein dairy cows. GPR54 has been reported in human breast cells [8], and the expression of GPR54 and Kp-10 has been detected in bovine primary placental cell lines isolated from cotyledons [9]. However, no reports have been published the characterization of GPR54 expression in the Holstein dairy cow mammary gland. In addition, we detected the gene and protein expressions of GPR54 in tissues from lactating Holstein dairy cows, revealing that the expression of GPR54 was higher in tissues from high-quality milk producers than in those from low-quality milk producers. The gene and protein expression of GPR54 exhibited a similar tendency toCSN2 in these mammary gland tissues. These results suggest that the activated GPR54 might be important in the production of CSN2. However, the functions of Kp-10 and GPR54 in bovine milk protein synthesis are not understood.

Prior studies have shown that the regulation of casein (including CSN2) is complex, and it is regulated by multiple hormones [37,38,39]. To elucidate the relationship between the synthesis of CSN2 and the activation of GPR54 in bovine mammary glands, we used Kp-10 to activate GPR54 in bMECs isolated from lactating Holstein dairy cows. The result showed that Kp-10 could increase the gene and protein expression of CSN2. Kp-10 could promote the synthesis of CSN2 by binding with GPR54. The bMECs were pre-treated with GPR54 antagonist P-234 before Kp-10 treatment, and the effect of Kp-10-induced CSN2 expression was blocked, supporting the role for GPR54 in regulating this process in bMECs.

**Figure 8:**
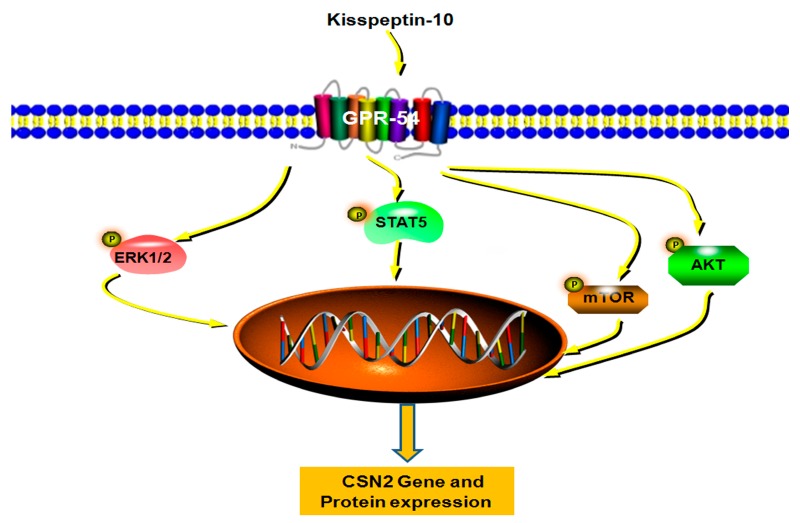
Schematic diagram of the proposed molecular mechanism for Kp-10 induce *CSN2* gene and protein expression in bMECs. Kp-10 binds to GPR54 and this leads to the activation of mTOR, AKT, ERK1/2 and STAT5 signaling pathways. The increased effect of CSN2 synthesis is blocked when the cells were pre-treated with the selective inhibitor of GPR54. All the inhibitors of the four signaling pathways attenuate the Kp-10-induced expression of CSN2 in bMECs. Altogether, Kp-10 facilitates the synthesis of CSN2 via GPR54 and its downstream signaling pathways mTOR, ERK1/2, STAT5 and AKT.

At present, we have found that Kp-10 can promote CSN2 synthesis by activating GPR54.However, the exact molecular mechanism remains unclear, and so the next step is to detect the changes in downstream signals in the process. The CSN2 synthesis process is likely to include several participating signaling pathways, including ERK1/2, AKT, STAT, mTOR [6,19,20,40,41]. In addition, Kp-10 plays the role of an anti-metastasis agent via β-arrestin2/ERK by binding with GPR54 in human breast cancer cells [8]. Furthermore, the activation of Kisspeptin-GPR54 signaling promotes the activation of nNOS through its phosphorylation via the AKT pathway in preoptic neurons in the study of the reproductive axis [42]. Moreover, mTOR signaling is closely related to the regulation of the *Kiss-1* gene at the onset of puberty [43]. In addition, the Kisspeptin expression is also regulated by the signal of STAT5 in the study of prolactin which causes suppression of Kisspeptin expression in the hypothalamus in mice [44]. Visible ERK1/2, AKT, and mTOR signals play an essential role in the study of *Kiss-1*/GPR54 signal. Thus, there may be some potential links between milk protein synthesis and Kisspeptin/GPR54. However, there were no relative reports about the link of Kisspeptin/GPR54 and CSN2 synthesis. In our study, we found that Kp-10 activated ERK1/2, AKT, mTOR and STAT5 pathways during the process of inducing CSN2 synthesis in bMECs. Further, the effect of Kp-10 on CSN2 synthesis in bMECs was blocked by pre-treatment with Peptide-234. In addition, the ERK1/2 inhibitor U0126, AKT inhibitor MK2206, mTOR inhibitor Rapamycin and STAT5 inhibitor AG490 attenuated the Kp10-induced increasing of CSN2. Therefore, the results indicated that Kp-10 increased the expression of CSN2 via GPR54 and the above four signaling pathways in bMECs. In conclusion, our study demonstrates that Kp-10 can induce the synthesis of CSN2 via its receptor GPR54, and the effect is mediated by mTOR, ERK1/2, STAT5 and AKT signaling pathways (Figure 8). Thus, the present findings may provide a certain theoretical basis or some treatment strategies for research and development in the dairy industry.

## 4. Materials and Methods

### 4.1. Reagents

Kp-10 was obtained from Tocris (Ellisville, MO, USA). DMEM-F12 (Dulbecco’s modified Eagle’s medium and Ham’s F-12 nutrient mixture) was obtained from Gibco (Grand Island, NY, USA). FBS (fetal bovine serum) was purchased from Clark (Richmond, VA, USA). Bovine insulin, prolactin, hydrocortisone, transferrin, epidermal growth factor, phosphatase inhibitor cocktail 2, protease inhibitor cocktail, and DAPI were all obtained from Sigma-Aldrich (St. Louis, MO, USA). The selective inhibitor of Peptide-234 was obtained from Tocris (Ellisville). The selective inhibitors U0126 (ERK1/2), MK2206 (AKT), Rapamycin (mTOR) and AG490 (STAT5) were purchased from Selleckchem (Houston, DE, USA). The Trizol was obtained from Invitrogen (Carlsbad, CA, USA), and the PrimeScript RT reagent kit with gDNA Eraser was purchased from Takara (Kyoto, Japan). The 2× Taq Master Mix was purchased from Vazyme (Nanjing, China). The SYBR Green QuantiTect RT-PCR Kit was obtained from Roche (South San Francisco, CA, USA). The Polybrene was obtained from Sangon Biotech Co., Ltd. (Shanghai, China), the RIPA lysis buffer from Beyotime Inst. Biotech (Beijing, China), and the PVDF membranes and ECL detection system from Millipore (Billerica, MA, USA).

### 4.2. Antibodies

Antibodies to p-ERK1/2 (Thr202/Tyr204), ERK1/2 (Thr202/Tyr204), AKT (Ser473), and p-AKT (Ser473), mTOR (Ser2448), p-mTOR (Ser2448), STAT5 (D3N2B) and p-STAT5 (D3N2B) were purchased from Cell Signaling Technology (Beverly, MA, USA). The antibody to CSN2 was purchased from Bioss Inc. (Beijing, China). The antibody to Kiss1-R (GPR54) was purchased from Proteintech (Wuhan, China). The antibodies for goat anti-rabbit IgG-HRP, rabbit anti-cytokeratin 18 (CK-18), and mouse anti-β-tubulin were purchased from Santa Cruz Biotechnology (Santa Cruz, CA, USA).

### 4.3. Ethics Statement

The study was supported and approved by the Animal Ethics Committee of Southwest University, and all experimental procedures and the care of the animals were in strict accordance with the “Guidelines on Ethical Treatment of Experimental Animals (20060227, No. 398)” issued by the Ministry of Science and Technology of China. Mammary tissue sampling described in detail below was performed under either general or regional anesthesia to minimize suffering and pain in the animals.

### 4.4. Tissue Acquisition, bMECs Culture and Treatments

Bovine epithelial cells (bMECs) in the mammary gland tissues from lactating Holstein cows (lactation = 120 ± 24 days) were isolated by the procedure previously described [45]. The isolated cells were cultured in DMEM/F12 medium (Irvine Scientific, Santa Ana, CA, USA), supplemented with 10% serum, penicillin (100 U/mL), streptomycin (100 U/mL), bovine insulin (5 μg/mL), and hydrocortisone (1 μg/mL), and prolactin (1 μg/mL), epidermal growth factor (EGF,10 ng/mL), and transferring (5 μg/mL). Immunofluorescence and flow cytometry were used to detect the expression of CK-18, which is a marker of bMECs. The gene expression of *CSN2* and *GPR54* was detected by PCR and Western blot in bMECs from passages 1–4.

Mammary tissue samples were obtained by a punch biopsy according to Baumgard et al. [46] with some modifications. Cows (lactation = 210 ± 24 days) were restrained from movement under general anesthesia. The tissues of high-quality milk group producers (feeding a low-concentrate mixed forage) and low-quality milk group producers (feeding a low-concentrate corn straw) were obtained from Inner Mongolia Agricultural University [47,48], As previously described [47], milk fat content (%) of the high-quality milk group (LCF) was up to 4.23 ± 0.69, and the low-quality milk group (LCS) was 3.95 ± 0.52; the milk protein yield (kg/day) and milk fat yield (kg/day) of LCF were 0.81 ± 0.13 and 1.07 ± 0.18, respectively, whereas the milk protein yield (kg/day) and milk fat yield (kg/day) of LCS were 0.51 ± 0.14 and 0.67 ± 0.22, respectively. The tissues were stored in liquid nitrogen for the subsequent experiments (*n* = 5 per treatment). The cells were treated with increasing concentrations of Kp-10 (0, 1, 10, 100 or 1000 nM) for 12 h after a 4 h starvation. The cells were then collected and frozen at −80 °C until mRNA or protein extraction. For some experiments, the cells were pre-incubated with inhibitors for 1 h, and then treated with Kp-10 for 12 h before the RNA and protein were extracted.

### 4.5. RNA Extraction, Reverse Transcription and Quantitative Real-Time PCR

The total RNA of bMECs and tissues was extracted using Trizol in accordance with the manufacturer’s instructions. The total RNA was stored at −80 °C quantified by measuring the absorbance at 260 nm and 280 nm. Each sample was transcribed to 4 μg total RNA. The extracted RNA was reverse transcribed using a PrimeScript reverse transcription Reagent Kit (Takara Bio, Shiga, Japan) with gDNA Eraser. The relative gene expression of*CSN2* with *β-tubulin* serving as the house keeping gene was evaluated by quantitative real-time PCR using the Premix Ex Taq real-time-PCR Kit, and each sample was repeated three times. The PCR products were visualized via 1.5% agarose gel electrophoresis. The primer sequences for the test gene are presented in Table 1.

### 4.6. Western Blot Analysis

A total of 2 mg each of the stored high-quality milk and the low-quality milk tissues stored in the liquid nitrogen were ground into a powder, and mixed with 50 μL RIPA lysis buffer. The cells were washed with ice-cold PBS and harvested using RIPA lysis buffer containing the following: 10 mM HEPES, pH 7.5; 10 mM KCl; 0.1 mM EGTA; 0.1 mM EDTA; phosphatase inhibitor cocktail 2, and a protease inhibitor cocktail. The resulting protein samples (30 μg) were subjected to 12% SDS-PAGE and transferred to a PVDF membrane. After blocking with 5% TBS-T milk, the membranes were incubated overnight at 4 °C with the specific antibodies to p-ERK1/2 (dilution 1:2000), ERK1/2 (dilution 1:2000), AKT (dilution 1:1000), p-AKT (dilution 1:1000), STAT5 (dilution 1:1000), p-STAT5 (dilution 1:1000), p-mTOR (dilution 1:1000), mTOR (dilution 1:1000), CSN2 (dilution 1:500), GPR54 (dilution 1:500), or β-tubulin (dilution 1:40,000). The membranes were washed four times with 0.1% TBS-T and incubated with goat anti-rabbit IgG-HRP or goat anti-mouse IgG-HRP for 1 h at RT. The proteins were visualized using an ECL detection system. 

### 4.7. Immunofluorescence Assay

The aim of the immunofluorescence assay was to identify the isolation of bMECs characteristic protein expression of CK-18. First, we seeded bMECs in a 24-well plate at 5 × 10^3^ cells per well. When the cell density reached 80%, the cells were washed with cold PBS and then soaked in fixative liquid for 15 min at RT, the cells were blocked with 5% donkey serum in PBS at 4 °C overnight. The next day, after being washed twice in PBS, the cells were incubated with the primary antibody against CK-18 (1:500) diluted in 5% donkey serum PBS at RT for 2 h. After being washed twice in PBS, the cells were incubated with PE-conjugated donkey anti-rabbit IgG (1:500) for 1 h at RT. After bMECs were washed three times, the cell nucleus was stained with DAPI for 15 min at RT. The end result was detected by inverted fluorescence microscope.

### 4.8. Flow Cytometry Assay

To test the purity of the cells, we used flow cytometry to detect the rate of occurrence of the characteristic protein CK-18 in bMECs. A total of 2 × 10^6^ cells per plate were seeded in 6 plates for 24 h. When the cell density was up to 80%, the cells were collected in 1.5 mL EP tubes, and then centrifuged and resuspended in 500 μL cold PBS. This washing procedure was repeated two or more times before the cells that were suspended in 500 μL pre-cooled fixative liquid and incubated for 1 h at RT. The cells were then washed twice with cold PBS, blocking with 5% donkey serum 100 μL at RT for 2 h. Discarding the sealing fluid (this step should not wash the cells), primary antibody CK-18 (1:100 diluted in 5% PBS donkey serum) was incubated with the cells at 4 °C overnight. The next day, after being washed with pre-cooled PBS, the cells were incubated with donkey anti-rabbit IgG-FITC at RT in the dark for 1 h. Finally, flow cytometry was used to detect the population of CK-18-expressing bMECs.

### 4.9. Statistical Analysis

All results are shown as the means ± S.D. All experiments were repeated three times at least, and each replicant was repeated three times or more. The data was analyzed using GraphPad Prism 5 (GraphPad InStat Software, San Diego, CA, USA).The comparisons between groups were carried out with an ANOVA followed by Dunnett’s test and the significance of the two groups of data were analyzed by Student’s *t*-test. The mammary gland tissues data is presented as the means ± S.D. (*n* = 5 per treatment), the bMECs mRNA and protein data is presented as the means ± S.D. (*n* = 3). We used *p* < 0.05 and *p* < 0.01 to represent data that was significantly different and extremely significantly different, respectively.

## Figures and Tables

**Table 1:** Sequences of the Primers Used to Amplify *GPR54*, *CSN2* and *β-tubulin*.

Gene	Sequences	Length (bp)
*GPR54*	(F) 5′-TGGCAATGCAGCCCTTGT-3′	149
	(R) 5′-GAAAAGCGGCACCAACCAG-3′	
*CSN2*	(F) 5′-AACAGCCTCCCACAAAC-3′	158
	(R) 5′-AGCCATAGCCTCCTTCAC-3′	
*β-tubulin*	(F) 5′-TCACCAACTGGGACGACA-3′	206
	(R) 5′-GCATACAGGGACAGCACA-3′	

## Abbreviations

Kp-10	Kisspeptin-10
CSN2	β-casein
BMECs	Bovine mammary epithelial cells
GPR54	Guanosine-binding protein coupled receptor 54
